# Concrete Made with Dune Sand: Overview of Fresh, Mechanical and Durability Properties

**DOI:** 10.3390/ma15176152

**Published:** 2022-09-05

**Authors:** Jawad Ahmad, Ali Majdi, Ahmed Farouk Deifalla, Hisham Jahangir Qureshi, Muhammad Umair Saleem, Shaker M. A. Qaidi, Mohammed A. El-Shorbagy

**Affiliations:** 1 Department of Civil Engineering, Military College of Engineering, Risalpur 4707, Pakistan; 2 Department of Building and Construction Technologies Engineering, Al-Mustaqbal University College, Hillah 51001, Iraq; 3 Structural Engineering Department, Faculty of Engineering and Technology, Future University in Egypt, New Cairo 11845, Egypt; 4 Department of Civil and Environmental Engineering, College of Engineering, King Faisal University, Al-Ahsa 31982, Saudi Arabia; 5 Service Stream Limited Co., Chatswood, NSW 2067, Australia; 6 Department of Civil Engineering, University of Duhok, Duhok 42001, Iraq; 7 Department of Mathematics, College of Science and Humanities in Al-Kharj, Prince Sattam bin Abdulaziz University, Al-Kharj 11942, Saudi Arabia

**Keywords:** dune sand, compressive strength, tensile strength, fine aggregate, durability

## Abstract

According to the authors’ best information, the majority of research focuses on other waste materials, such as recycling industrial waste (glass, silica fume, marble and waste foundry sand), etc. However, some researchers suggest dune sand as an alternative material for concrete production, but knowledge is still scarce. Therefore, a comprehensive review is required on dune sand to evaluate its current progress as well as its effects on the strength and durability properties of concrete. The review presents detailed literature on dune sand in concrete. The important characteristics of concrete such as slump, compressive, flexural, cracking behaviors, density, water absorption and sulfate resistance were considered for analysis. Results indicate that dune sand can be used in concrete up to 40% without any negative effect on strength and durability. The negative impact of dune sand on strength and durability was due to poor grading and fineness, which restricts the complete (100%) substation of dune sand. Furthermore, a decrease in flowability was observed. Finally, the review highlights the research gap for future studies.

## 1. Introduction

All construction and development initiatives require concrete as their base since it is a widely used building material worldwide [1,2,3,4]. Finding lower priced cement made from local natural resources has emerged as a primary objective in order to make up for the scarcity in cement manufacturing [3,5,6,7]. Since 1970, there has been a great deal of research on the application of cementing additives as a partial substitute for Portland cement. The byproducts of other industry or natural sources are these additions [8]. The practical binding activity that the supply of the additive is influenced is by the volume, fineness, mineralogical composition and kind of cement, which in turn enhances the strength [9]. The density of the mortar might be raised by the creation of the secondary cementitious material calcium silicate hydrates (CSH). The latter is produced by including minute siliceous particles that serve a particular pozzolanic purpose and increase the strength properties of concretes [10,11,12].

Each of the most important ingredients of concrete has an impact on the environment to varied degrees. Numerous sustainability issues are brought up by the massive amounts of concrete utilized globally [13,14,15]. There has been considerable concern due to a rise in the amount of riverbed sand and gravel used in concrete [16]. Due to the extensive usage of concrete brought on by the surge in urbanization and industrialization, more natural sand has been removed from riverbeds. A few of the negative effects include increased riverbed distance, a drop in the water table, the discovery of bridge substructures, a significant impact on rivers, deltas, coastal ecosystems and marine ecosystems, land loss due to river or coastal erosion and a reduction in the quantity of deposit sources [17]. Furthermore, limits on sand removal from a river, which have led to an increase in sand charges, have seriously impacted the ability of the building sector to survive [18].

One of the essential ingredients for producing mortar and concrete is a fine aggregate, which also plays a crucial part in the design mix [19]. Fine aggregate is a key component of concrete, and the amount and kind of sand used to create a particular concrete mix will define its qualities. It significantly affects the flowability, resiliency to the effects of the environment, strength and dry shrinkage of concrete. In comparison to cement, sand makes up a bigger portion of the mixture. Another element that adds to concrete strength is that sand may fill up any pores or spaces in the material. Sand offers a mass of particles that can resist the action of applied stresses and endure longer than mortar alone. Fine aggregate also lowers volume changes brought on by the setting and hardening processes. Sand is thus essential for concrete’s capacity to consolidate and give the necessary strength. In place of natural sand, there are other alternate sand options, such as foundry sand [20], marble waste [21], waste glass [22], recycled concrete aggregate [23], recycled ceramics aggregate [24], copper slag [25], iron ore tailing [26], plastic waste [27] and crush stone dust [28] that may be utilized as sand in concrete.

It is generally known that many construction projects, including building construction in recent years, water delivery channels, oil exploitation platforms, and building construction, are to be constructed in the desert. River sand is a crucial primary component for the increasing infrastructure projects. However, owing to the great distance and overuse of the river channel in several desert areas, the decline in river sand and the rise in need have come to be an insurmountable challenge. Despite being widely distributed across the desert, dune sand is seldom used in engineering construction, owing to its high prevalence of very small particle sizes and inability to conform to particle size grading standards.

Researchers utilize clay to make cement clay mortar as a masonry binder to construct buildings, reducing the need for cement, saving money and protecting the environment [29]. If dune sand and clay, which are effortlessly obtained nearby, can be technically utilized in place of natural fine aggregate and a binder as a building material, that will not only satisfy the demand for infrastructure creation, but also significantly reduce the charges of construction projects, making waste profitable. Many findings have been reported on the use of dune sand as a fine aggregate to create concrete, particularly in desert regions, in order to satisfy the criteria of engineering construction utilizing local supplies and decreasing the shipping expenses [30,31].

In light of these conditions, there is growing interest worldwide in the idea of substituting dune sand and clay for natural fine aggregate and cement in the creation of concrete and mortar. Some scholars have recently looked at the characteristics of mortar and concrete. The rheological characteristics of mortar including crushed sand were investigated by Mikael et al. [32]. The effect of natural river sand on the properties of mortar containing suspensions of coarse particles was assessed. According to the experiments, fine aggregate has a significant impact on both the water need and the mortar usability. The features of concrete made from dune sand from Maowusu sandy terrain have been studied by Jin et al. [30].

The issue of cementitious materials created using dune sand has sparked growing attention because of the environmental advantages. Numerous studies on the features of dune sand and the qualities of cementitious materials using it have been performed over the last ten years. Dune sand differs from regular engineering sand in that it has a poor gradation and high salinity. The varied mechanical characteristics of cementitious composites are considerably impacted by a high replacement rate of dune sand for construction sand [33]. The concrete made with a dune sand to fine aggregate ratio of 10% showed the highest compressive strength [34]. By adding ultrafine fibers [35] and mineral fillers [36] to cementitious composites or by regulating fractures and permeability utilizing ultrahigh performance materials at the macro level, the strength of dune sand-integrated cementitious composites may be increased.

The impact of very fine particles on the flowability and strength of concrete created with dune sand from the Australian desert was studied by Fu Jia Luo et al. [37]. The findings revealed that the metal fibers alter concrete properties by various mechanisms depending on the concentration of the sand–cement ratio and have no adverse effects on flowability. Additionally, dune sand concrete strength is on par with or even exceeds that of natural fine aggregate concrete. By valuing dune sand and pneumatic waste metal fibers, Allaoua Belferrag et al. [38] researched how to improve the compressive capacity of cement in dry conditions. The impact of adding a novel class of metal fibers made from recycled tires on the compressive strength of dune sand concrete has been researched. In comparison to concrete without fibers, the findings obtained reveal an increase in the compressive capacity for metal fiber-reinforced sand dune concrete. According to Krobba et al. [39], the characteristics of mortar, including density, strength, shrinkage, elasticity and bonding strength, are affected by the addition of natural microfibers. In comparison to mortar made from beach sand (without Alfa natural microfibers) and tested under identical circumstances, the findings obtained demonstrated that mortar containing natural microfibers had improved mechanical and physical qualities [39].

**Figure 1:**
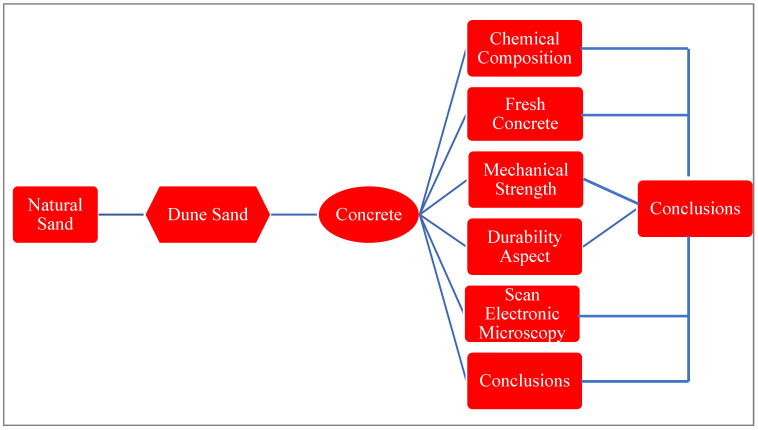
Different sections of the review.

Brief literature shows that dune sand can be used as an alternative material for the production of concrete. However, knowledge is still scarce, which restricts the use of dune sand in concrete, and a comprehensive review is required to identify the positive and negative effects of dune sand on concrete performance. Furthermore, according to the authors’ best information, no detailed review on dune sand concrete has been considered up to now. Therefore, this review presents the detailed literature on the characteristics of concrete with dune sand. For analysis, the important properties of concrete such as slump, compressive strength, flexural strength, cracking behaviors, density, water absorption and sulfate resistance were taken into account. The successful review provides multiple benefits, including the current progress of dune sand in concrete, the effect of dune sand flowability, strength and durability performance of concrete. The review also suggests future guidelines for the upcoming generation. Figure 1 represents a different section of the review.

## 2. Physical and Chemical Properties

Dune sand is very fine sand with a maximum particle size of 1.18 mm [40]. The diameters of more than 90% of its components are between 0 and 0.4 mm [41]. Dune sand typically has a fineness modulus of less than 1.5 or average particle size of less than 0.25 mm, making it superfine sand [42]. Dune sand is classified as a D1 soil type by the GTR 2000 Soil Classification [43]. It is described as porous, irregular and poorly graded dirt. This implies that dune sand by itself will not be adequately compact, and as a result, its immediate bearing index is insufficient [44]. Therefore, it will be crucial to treat this sand using hydraulic binders as correctors.

The largest size of the coarse grains is 0.5 mm, while the diameter of the tiniest particles is in the range of 0.04 mm. It is fine golden sand. The curvature coefficient (Cc) is around 0.96, while the uniformity coefficient (Cu) is in the range of 2.0. Therefore, it is extremely fine, improperly graded sand. The most often calculated component for fine aggregates is the fineness modulus, which is used to find the aggregate gradation degree of consistency. The examined dune sand samples had fineness modulus values ranging from 1.07 to 1.30. These findings show that the investigated dune sands fall short of the fine aggregate gradation criteria. Therefore, to achieve an appropriate degree of gradation, it is important to increase the gradation of these dune sands by combining them with well-graded crushed fine aggregates of ceramic waste [45]. Furthermore, physical properties are presented in Table 1.

**Figure 2:**
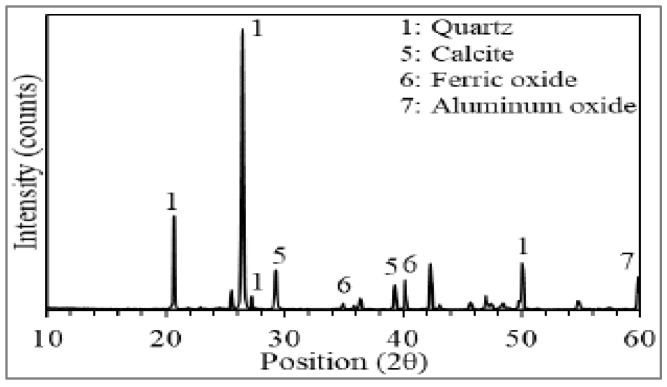
XRD of dune sand [51].

Dune sand is siliceous, made up mostly of SiO_2_ (silica), with traces of calcium and magnesium species, according to the chemical analyses reported in Table 2. X-ray diffraction of the dune is shown in Figure 2. High percentages of quartz and traces of illite and calcite are found, according to X-ray examination [50].

## 3. Slump Flow

Concrete workability refers to how easily new concrete may be poured, reinforced and finished, with the fewest uniformity losses possible. Any concrete mixture should be workable enough to be properly poured, solidified and filled into the forms, as well as to encircle any embedded reinforcement or other objects.

**Figure 3:**
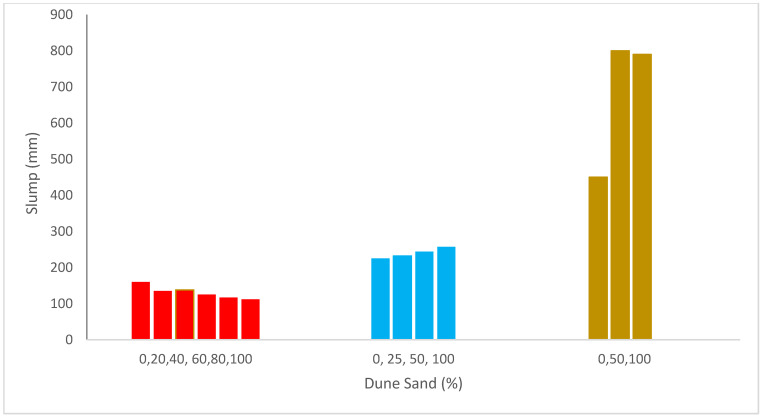
Slump flow: data source [40,48,58].

Figure 3 shows the slump flow of concrete with the substitution of dune sand. Dawood et al. [40] reported that the slump flow of concrete decreased with the addition of dune sand. The dune sand particle size distribution is mostly responsible for the decline in a slump. It is well-known that when the fines particles content rises, the flowability of concrete declines as well [54]. The lack of chemicals in the concrete resulted in poor workability and a minor slump [55]. As a more natural aggregate was substituted with recycled concrete aggregate (RCA), the densities and slump of fresh concrete reduced. Although the slump was further decreased, the addition of steel fibers increased the densities of both fresh and cured concrete [46]. Increases in dune sand concentration lowered flowability from a high to medium level by 30%, and the addition of steel fibers also dramatically decreased workability [40].

A dramatic decline in a slump was seen in all mixtures, producing concrete with a very low slump when all the fine particles were completely substituted by beach sand (100 percent substitute). When more regular sand is substituted with dune sand, the particle size distribution that results causes the slump to diminish. It is common knowledge that concrete becomes less workable as fine particles are used [56]. The decrease in slump flow due to dune sand may be due to the fact that the mixing water may be found as free layer water, adsorbed layer water and filled water, according to Wang et al. [57]. The many types of water each contribute differently to workability. The solid particles are separated from one another by the free layer of water, which improves workability. The water-adsorbed layer is quite near the solid’s surface. This portion of the water will be constrained by the solid particle and unable to flow freely as a result of the solid surface adhering to the water molecule. Therefore, this water has no effect on workability. The filling water does not affect the workability; it only fills the spaces between solid particles. It should be noted that the kind and quantity of the various solid particles determine the types of water that may interchange in the system. For instance, the fine grains will fill the holes of the granular structure by distributing the water in these holes when the right ratio of fine and coarse particles is combined together.

On the other hand, Figure 3 shows that the slump rises as the dune sand content rises, according to Rennani et al. and Leila et al. [48,58]. The spherical form of the dune sand particles, all things being equal, is thought to be responsible for the rise in a slump. This is because spheres travel more easily than angular or awkward-shaped particles because of the ball-bearing effect. The slump did, however, start to subside with dune sand concentrations over 50%. All findings indicate that despite an increase in beach sand content, concrete may still be made to be workable. Additionally, it was found that the amount of dune sands the mixture could contain before slumping down increased with the workability of the mixture.

## 4. Mechanical Strength

### 4.1. Compressive Strength

**Figure 4:**
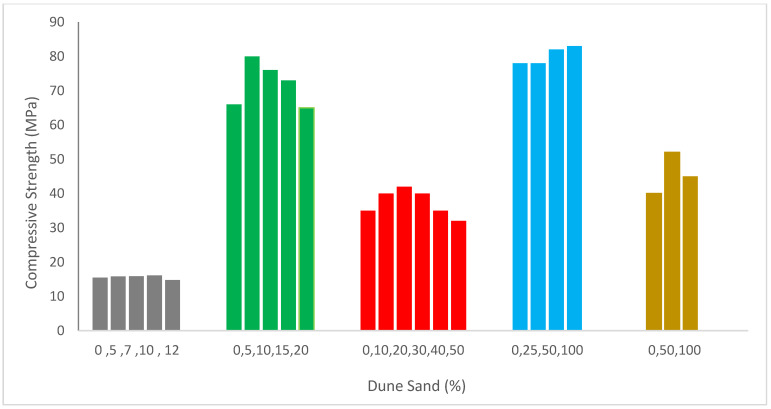
Compressive strength: data source [10,47,48,49,58].

Figure 4 shows concrete compressive capacity with dune sand’s substitution. It can be noted that the compressive capacity of concrete was enhanced up to a certain extent by the substitution of dune sand and then decreased. According to certain researchers [56,59], when the dune sand replacement ratio rose, concrete’s compressive capacity decreased. Due to the dune sand particles’ smooth surfaces and rounded shapes, the binding strength between the cement paste and dune sand may have been reduced [60]. In contrast, several studies [61,62] found that the strength improved to a specific replacement ratio for the dune sand but declined beyond that point. According to Luo et al. [59], high aggregate compactness might cause a rise in strength. According to previous research, dune sand may affect the compactness of aggregates and the strength of the link between dune sand and cement paste. Additionally, the impact of dune sand on each side has a different effect on compressive strength.

It was found that replacing 40% and 50% of the natural dune sand with waste ceramic aggregate resulted in the biggest gain in compressive and flexural capacity when compared to reference mortar. At a 40% and 50% mix ratio of ceramic under various curing conditions, the compressive strength of dune sand ceramic mortar achieves excellent strength [45]. The findings show that as dune sand content rises, concrete strength typically declines. As fine particles have a larger surface area, more grout is required to cover their surface, which results in a loss in strength [56].

The compressive capacity of mortar improves initially with an increase in the dune sand replacement ratio before decreasing and peaking at a 20 percent replacing ratio [47]. According to Appa Rao [11], at a constant water/binder ratio of 0.5, adding silica fume to a mortar up to the point where it replaces 30 percent of the cement results in an increase in compressive strengths [63]. These findings reveal that the development of compressive capacity as a function of time (7, 28 and 90 days) reveals that the compressive capacity is low for all samples during the first seven days but then dramatically rise over the succeeding periods. This is caused by the kinetics of the time-dependent reactions between dune sand powder and portlandite and the hydration of cement [10]. In accordance with the water/binder ratio, Kwan [64] demonstrated that adding silica fume to a mortar with up to 15% of the cement replacement amount results in an increase in compressive strengths after 28 days [65]. Based on our findings, we can conclude that adding the fiber and applying a heat treatment boosted the compressive and flexural capacity. The quantity of dune sand rose, and the compressive strength somewhat improved [66]. Linear connections between compressive capacity and fine dune sand % with respectable correlation coefficients demonstrate that compressive capacity rises as dune sand percentage increases [48]. The findings suggest that when the rate of dune sand replacement increases, the compressive capacity of specimens initially rises and subsequently falls. At curing ages of 7 days and 28 days, respectively, the compressive capacity of the specimen including 50% dune sand improves by 3.87 percent and 10.64 percent compared to the specimen containing 0% dune sand.

The compressive capacity increased the percentage of dune sand. However, the highest strength loss was just around 25% [56]. According to a study, concrete’s compressive strength declines by 39% when the substitute ratio reaches 100%, but it increases with the addition of single or hybrid fibers. In contrast, sand dunes have a negative impact on compressive capacity, particularly when the proportion increases by more than 40% [40]. The research has shown that dune sand may be utilized as an affordable and easily accessible substitute for sea sand and can therefore assist in stopping the negative impacts on the ecosystem caused by excessive sea sand mining [67].

A study reported no significant changes in the amount of addition or dune sand percent on the setting time of mixes. The pastes’ original consistency was dramatically reduced by the addition of dune sand. Due to their higher fineness than that of ordinary Portland cement (OPC), dune sand powder acts as a lubricant by reducing the intergranular vacuum. This cement has a lot of vacuums that need to be filled with water before they can set in the convenience of the mortar. Therefore, in order to decrease the intergranular vacuum and hence the need for water in cement, it is required to enhance the quantities of tiny and big particles. The intergranular vacuums will be filled with the dune sand powder, reducing the need for water [10].

**Figure 5:**
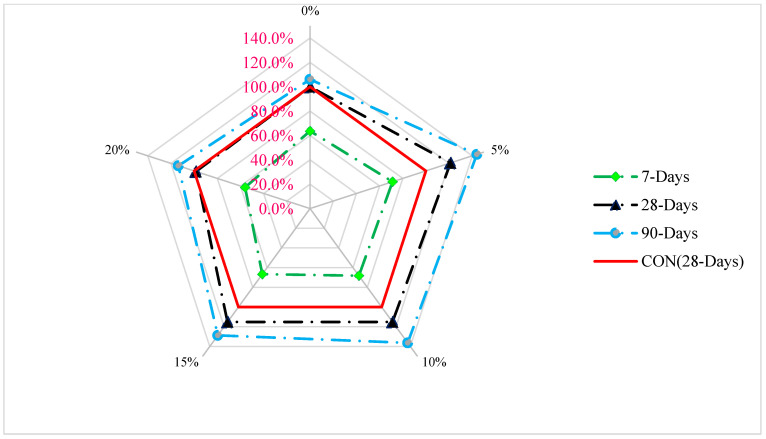
Compressive strength age relation [10].

A comparative investigation of compressive capacity with different dune sand dosages at various curing days is shown in Figure 5.

As a reference mix, the compressive capacity of control concrete on day 28 of curing was used to evaluate the compressive strength of different dosages of dune sand at various curing days. For a comparative study, five percent of the recommended dune sand dosage was considered. Compressive strength during seven days of curing is 29% less than the reference compressive strength (concrete at 28 days controls compressive capacity with a 5% replacement of dune sand). The compressive strength with a 5% substitute of dune sand is 21% higher than the reference concrete’s compressive strength after 28 days of curing. Compressive capacity is 43% more than the reference concrete at the same dosage of 5% dune sand replacement. Additionally, with a 20 percent replacement of dune sand, the compressive strength at 28 days is the same as the reference concrete’s compressive capacity, but by 90 days, it is 14 percent higher than the reference strength.

### 4.2. Tensile Strength

**Figure 6:**
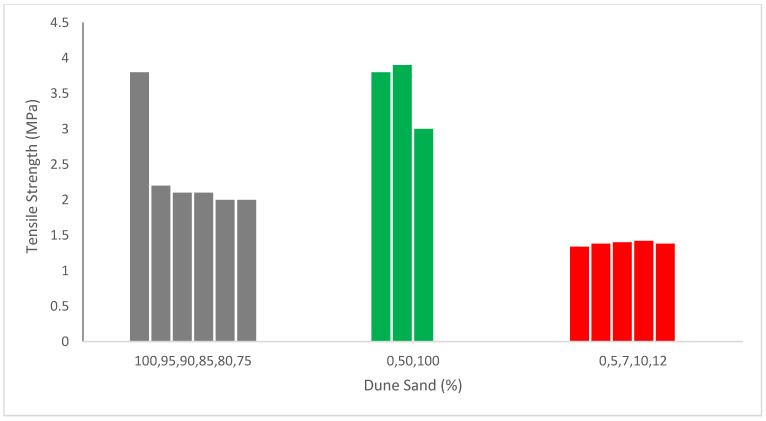
Tensile strength: data source [49,58,68].

Figure 6 illustrates the tensile capacity of concrete with the substitution of dune sand. It can be noted that the tensile capacity of concrete reduced with the substitution of dune sand as reported by Abadou et al. [68].

The modulus of elasticity and tensile capacity of concrete is not significantly negatively impacted by increasing the amount of dune sand in concrete [56]. The DS/FA (Dune sand/Fly ash) ratio of 10% was ideal for compressive and tensile capacity under the mixed circumstances of this investigation. The strength differential might be more than 10%, depending on the DS/FA ratio [34]. Since the compressive capacity has greatly increased and the tensile strengths have only marginally diminished, it was possible to create a material that may be considered when constructing pavement constructions by adding dune sand and lime to permeable asphalt [49]. At all evaluated ages up to 44 percent, the recycled aggregate made from concrete waste used to make the dune sand mortar had lower tensile strength than the specimen mortar. When compared to ordinary mortar, the integration of concrete waste aggregates does not seem to have a substantial influence on tensile strength [68]. The tensile and flexural capacity decreased by 29.3 percent and 21.1 percent for splitting and flexural capacity, respectively, and at a slower pace than the compressive strength as the sand dune rate increased. The ratio (40 percent DS without fibers) attained the greatest tensile capacity of 4 MPa (22.3 percent more than the reference samples), according to the tensile capacity data at the age of 28 days.

On the other hand, Figure 6 shows that the tensile capacity of concrete rises as the dune sand content rises, according to Rennani et al. and Leila et al. [48,58]. Similarly, 60 percent dune sand increased tensile strength by 0.91 percent, which is equal to the reference concrete. The mechanical qualities of the hybrid concrete were enhanced by the inclusion of steel fibers, which can make up for much of the lost strength caused by the rise in sand dunes ratios [40]. The tensile strength of the hybrid mixture is 40.2 percent higher than 100 percent dune sand without fibers when dune sand is completely replaced by 100 percent rather than sand with 1 percent fibers [40]. Fiber increased tensile strength due to the prevention of cracks [69]. Even if the cracks appear, the propagation of cracks is restricted by the fibers [70].

### 4.3. Flexural Strength

**Figure 7:**
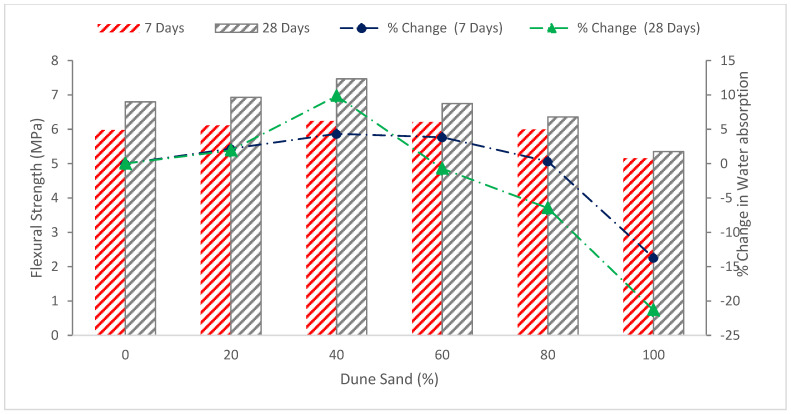
Flexural strength: data source [40].

Figure 7 reveals the flexural capacity of concrete with the substitution of dune sand. It can be noted that flexural capacity improved by up to 20% with substitution of dune sand. A study reported that the flexural strengths were increased by using waste materials as aggregate. It was found that replacing 40% and 50% of the natural dune sand with waste ceramic aggregate resulted in the biggest gain in compressive and flexural strength when compared to reference mortar [45]. According to the experimental findings, mechanical properties were best when dune sand replacement was 50%. The flexural strength of self-compacting mortar had not been significantly decreased by the replacement of dune sand [54].

The 40% substitution of dune had the maximum flexural strength at seven days, which is 4.3 percent higher than the reference sample. At the same time, the flexural capacity is reduced by 13.7 percent less than the reference specimen when dune sand is substituted 100 percent (completely replaced the sand). The 40 percent dune sand in concrete shows the maximum flexural strength (7.47 MPa) at the age of 28 days, which is 9.85 percent higher than the reference sample. When the dune sand ratio is enhanced, the flexural capacity decreases. At a replacement ratio of 100 percent dune, flexural strength decreased by 21.3 percent when associated to the blank concrete (without dune sand). The rise in fineness of the sand particles in the concrete is the cause of the decreased flexural capacity. However, the flexural capacity of concrete with dune sand substituted was significantly enhanced with the addition of fibers. A concrete mixture of 20% dune sand along with 1% steel fibers achieved the highest flexural capacity of 10.07 MPa at the age of 28 days, which is 45.3 percent higher than the reference concrete [40]. Few researchers considered flexural strength in their studies. Therefore, a more detailed investigation is required.

**Figure 8:**
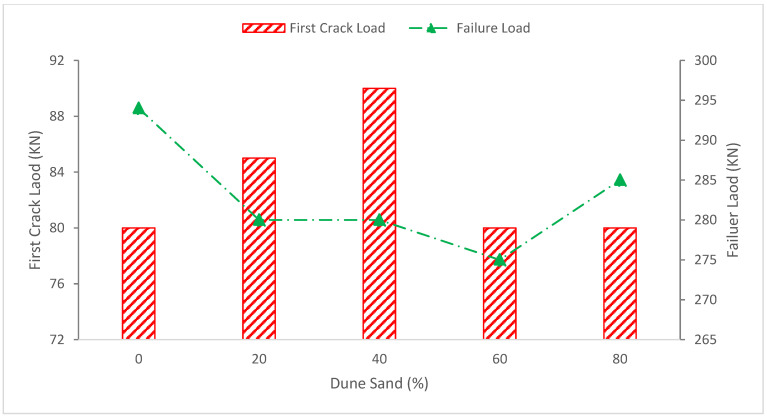
First crack load and failure load: data source [71].

Flexural and flexure–shear fractures are clearly visible, and the crack patterns of the regular beam resemble those of dune sand beams. It was determined that the presence of the dune may have delayed the initial fracture since it occurred at a force of around 80 KN, as shown in Figure 8, the central flexural zone. Shear fractures began to appear in both shear spans at the supports at a load of around 130 KN, although their dispersion in the shear spans varied. All beams broke in flexure shear at a load of around 280 KN, and the ultimate failure took place near the load point for concrete crushing in the compression zone. Figure 8 shows the failure load for every beam, and it can be inferred that the presence of dune sand may lower the failure load of beams. Although the leading shear crack direction and failure mechanisms varied, the initial fracture, failure load and shear cracks did not.

## 5. Durability

### 5.1. Dry Density

**Figure 9:**
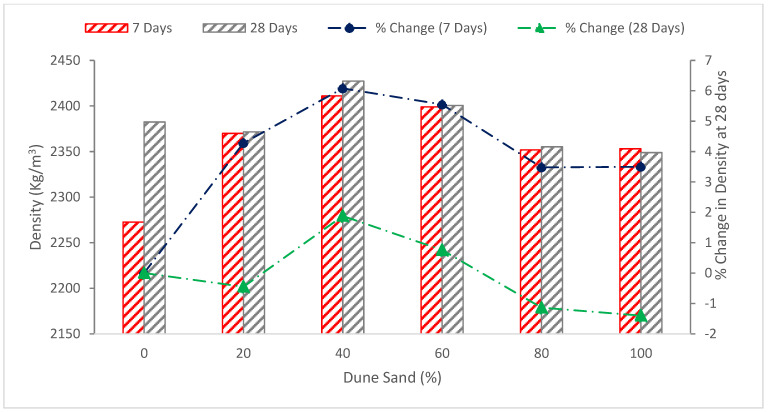
Dry density: data source [40].

The addition of dune sand to the concrete mixture increased the density at replacement ratios of 40 and 60 percent, with the replacement ratio of 40 percent dune sand. The recording density is 2427.35 kg/m^3^ with a growth of its quantity 1.88 percent more than the blank concrete, while the replacement ratio is 60 percent, and the increase in density is 0.76%, as associated with the control concrete. Partial substitution of dune with natural sand helped increase the density due to the softness of the dune sand, their spherical form and their overlap between gravel and sand grains, which helped fill all the spaces and holes within the concrete [40]. A study also reported that the filler materials increased the density of concrete due to filling cavities in concrete components, leading to more dense concrete [72]. However, when the percentage of dune sand is raised to 80% and 100%, the density falls, as shown in Figure 9. It might be possible that a higher dose of dune sand reduced the density due to a lack of flowability. The less flowable concrete required more compaction energy as compared to more workable concrete. Therefore, more chance of voids in less workable concrete, which adversely affect the density of concrete. A study also claims that filler material decreased the density of concrete at a higher substitution ratio due to lack of flowability [19].

### 5.2. Water Absorption

**Figure 10:**
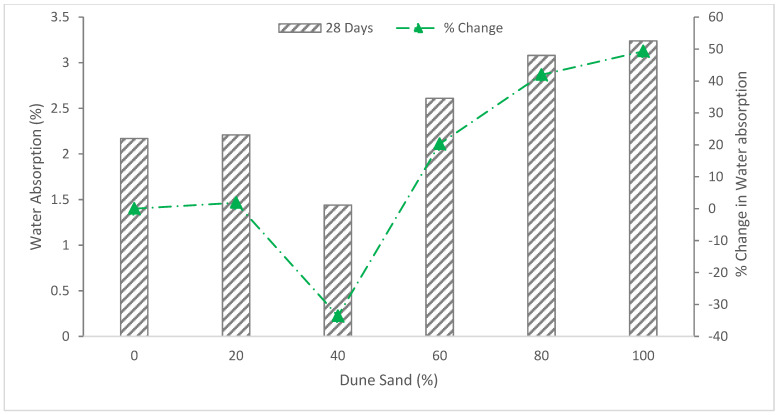
Water absorption: data source [40].

Figure 10 shows the water absorption of concrete with the substitution of dune sand as fine aggregate at 28 days.

The 40% substitution of dune sand with fine aggregate shows the lowest water absorption rate of 1.44 percent, which is 33.6 percent less than the reference mixture. The decrease in water absorption is due to the micro-filling voids effect of dune sand, which fill the voids, leading to more dense concrete. According to CEB 1989;192, this specimen is regarded as excellent concrete because of its low water absorption. The rate at which water is absorbed increases when the dune sand concentration rises by 60%, 80%, and 100%. The absorption rate at 80% and 100% substitution of dune sand are 41.9 and 49.3 percent, respectively, which is more than the reference mixture, and the concrete quality is medium, according to CEB 1989;192. A similar finding is also reported by other researchers that the quality of concrete decreased at a higher substitution ratio of dune sand [56]. It could be caused by the fineness of the dune sand grains, which absorb more mixing water and have a larger surface area, creating holes or spaces that, in turn, speed up the rate of absorption.

### 5.3. Ultrasonic Pulse Velocity (UPV)

**Figure 11:**
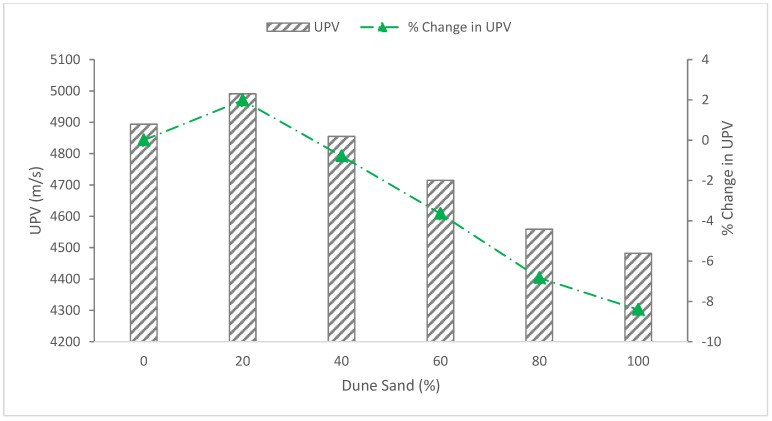
Ultrasonic pulse velocity (UPV): data source [40].

The findings showed that all concrete mixes had pulse velocities between 4400 and 6100 m/s, which is consistent with excellent and homogenous concrete quality as defined by BS1881, 1983, Part 116. The reference specimen shows a pulse velocity rate of 4894 m/s, and the concrete quality satisfies the requirements of IS code BS1881, 1983. As shown in Figure 11, the pulsing velocity for a concrete mixture of 20% dune sand is 1.98 percent higher than that of the reference specimen (0% dune sand), and it decreases with increasing dune sand content for the ratios 40%, 60%, 80% and 100%. This is consistent with the absorption and density test results, which also show a decrease with increasing dune sand content due to the fineness of the dune [40].

### 5.4. Sulfate Resistance

#### 5.4.1. Visual Observation

**Figure 12:**
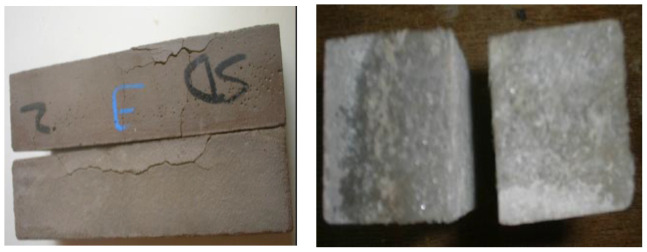
Beam of dune sand mortars immersed in sodium sulfate solution (5%) at 180 days [73].

Every month, a comprehensive visual inspection was conducted on the mortar specimens subjected to sulfate assault to assess any evident symptoms of softening, cracking and spalling. Figure 12 depicts typical instances of the harm that sulfate assault on mortar specimens causes after 180 days of immersion in sodium sulfate. It was found that the mortar samples’ corners always showed the first signs of degradation, and the broad cracks caused by the expansion strain on the cement matrix were substantial, as shown in Figure 12. After 180 days of immersion, the dune sand mortar began to visually deteriorate as a result of the mortar structure losing its cohesion. Additionally, a layer of white material was discovered deposited on the mortar faces, which was verified by the XRD investigation to be gypsum and ettringite formation.

#### 5.4.2. X-ray Diffraction (XRD)

**Figure 13:**
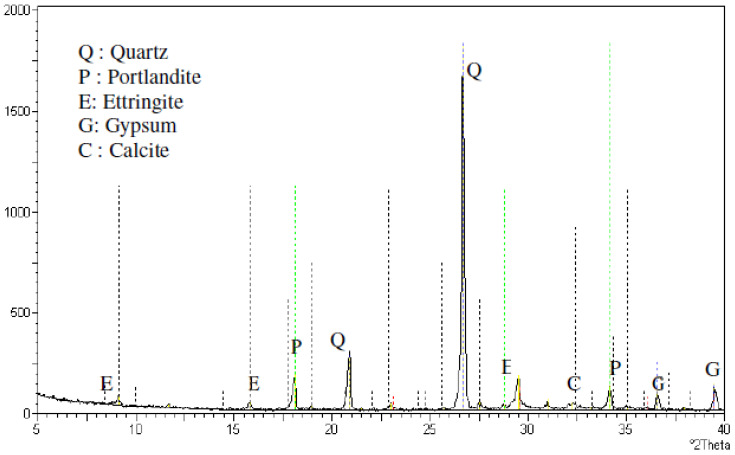
XRD of dune sand mortars immersed in sodium sulfate solution (5%) at 180 days [73].

Figure 13 shows the X-ray Diffraction (XRD) examination of mortar that was exposed to a five percent sodium sulfate solution for 180 days, which shows diffractograms demonstrating the gypsum-specific peaks. On the degraded portions of cube mortar samples that were evaluated for compressive strength, the XRD analysis was performed. It can be seen that some ettringite peaks were found as a result of sulfate assault, together with calcite. Peaks made of quartz and portlandite (made from sand) were visible. The mortars for all showed gypsum and ettringite are two common sulfate attack byproducts that lead to mortar strength loss [74].

#### 5.4.3. Strength (Compressive and Flexural)

**Figure 14:**
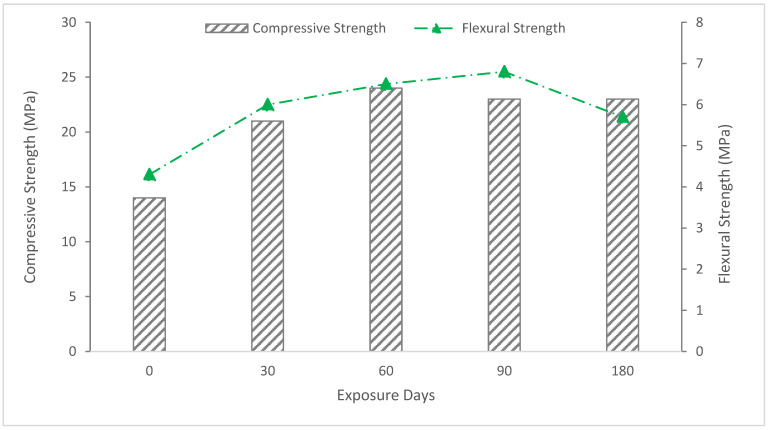
Strength of dune sand mortars immersed in sodium sulfate solution (5%) at 180 days [73].

Strength changes in mortar specimens subjected to sodium sulfate solution (5%) are shown in Figure 14. It can be seen that all mortars demonstrate a strength increase at the beginning of the exposure duration and a subsequent decline in strength development. Strength initially increased in sulfate solution as a result of additional hydration products filling the pores, but strength afterward reduced as a result of microcracking caused by expanding components of sulfate assault. After 180 days of exposure, mortar exhibited a quick loss of strength, and the loss of strength for dune sand mortar was considerable.

## 6. Scanning Electron Microscopy

**Figure 15:**
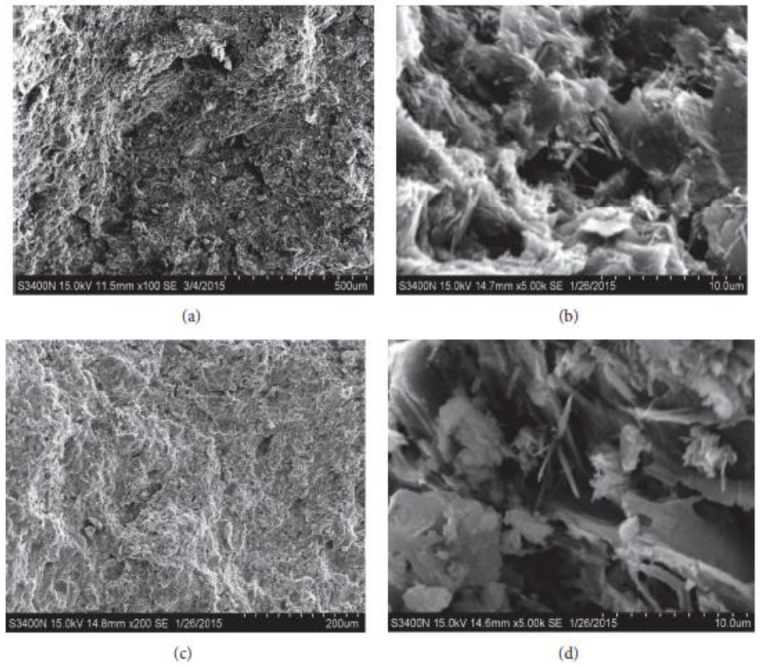
SEM Results (**a**,**b**) 7 days and (**c**,**d**) 28 days [53].

The hydration process of cementitious materials (ground granulated blast furnace slag) generated using dune sand was explained using the scan electronic microscopy (SEM) method. Figure 15 displays SEM pictures of the microstructure at the 7- and 28-day curing ages. Figure 15 shows that the cementitious material’s microstructure is highly thick, particularly after 28 days, which may be explained by the significant decrease in void regions brought on by hydration processes. The existence of different cementation compounds in the dune sand matrix was indicated by several hydration products such as C-S-H gel, Ca (OH)_2_, and ettringite at the matrix, which also confirmed the development of cementitious compounds. The development of strength is significantly impacted by these hydrated chemicals. At seven days, tabular Ca (OH)_2_, cotton-shaped C-S-H gels, needle ettringite and their interactions with one another produced a stable paste structure. At 28 days, the pores are completely filled with the C-S-H gel and ettringite, which progressively corrects the structural flaws. Structures become denser with time in comparison to early specimens, which indicates that the specimen’s strength grows.

## 7. Conclusions

The review focused on the use of dune sand as a fine aggregate in the manufacturing of concrete. The physical and chemical composition of dune sand and fresh, strength and durability characteristics of concrete were reviewed and compared. Although using dune sand as a greater or complete replacement for natural sand in the making of concrete has certain negative effects on the performance of the concrete, it maybe used in the creation of concrete to a limited degree. The best substitute dosage for the majority of the attributes examined has been found to be 30 to 40 percent. A detailed conclusion is provided below.

- The physical property of dune depicts that the fineness modulus of dune sand is much lower than river sand. Additionally, a poorly graded and irregular shape adversely affects the flowability of concrete.
- The sum of different chemicals such as silica, iron, lime, alumina and magnesia are greater than 70%. Therefore, it might be possible to use cementitious materials or cement ingredients during the manufacturing of cement.
- Slump decreased with the substitution of dune sand due to its physical nature (rough surface and poorly graded).
- Mechanical strength such as compressive, flexural and tensile capacity is improved to some extent. However, a higher dose or complete substitution adversely affects strength properties. The optimum dose of dune sand varies from 30 to 40%.
- Durability properties such as water absorption, density and sulfate resistance improved with dune sand, but less information is available.

## 8. Future Studies Recommendation

- The overall studies demonstrate that dune sand has the credibility to be used in concrete. However, the following aspects should be studied before being used in practice.
- The highest strength loss was just around 25%. Therefore, this decline was quite small and can be improved by adding fibers or other pozzolanic materials, such as fly ash and silica fume, waste glass, etc.
- Detailed investigations on durability performance should be explored.
- Dune sand as a cement ingredient in the manufacturing of cement should be explored.
- Treatment of dune sand before use, such as heat or alkaline solution, should be explored.

## Figures and Tables

**Table 1:** Physical properties of dune sand.

Authors	[45]	[46]	[47]	[48]	[49]
Dry-rodded density kg/m^3^	-	1663	-	-	-
Water Absorption (%)	-	-	3.84	-	-
Fineness Modulus	1.07	1.45	1.44	-	0.65
Sand equivalency (%)	87%	-	-	-	69%
Bulk density (kg/m^3^)	2525	-	1560	-	-
Specific Gravity	-	2.77	-	-	2.64
Surface area cm^2^/g	-	116.8	-	3000	-
Apparent specific gravity (kg/m^3^)	1434	-	-	-	-

**Table 2:** Chemical Composition of Dune Sand.

Authors	[10]	[50]	[51]	[52]	[53]
SiO_2_	74.61	93.56	64.9	64.58	65.63
Al_2_O_3_	1.35	1	3.0	9.48	12.63
Fe_2_O_3_	0.86	1	0.7	2.32	3.42
MgO	0.29	-	1.3	2.06	4.52
CaO	17.3	-	14.1	8.62	8.76
Na_2_O	-	-	-	2.43	1.83
K_2_O	0.47	-	-	1.97	1.87

## Data Availability

All the data available in main text.
